# Trends and Outcomes of Proton Radiation Therapy Use for Non–Small Cell Lung Cancer

**DOI:** 10.14338/IJPT/18-00029.1

**Published:** 2018-11-30

**Authors:** Amy C. Moreno, Ning Zhang, Sharon H. Giordano, Zhongxing Liao, Daniel Gomez, Joe Y. Chang, Steven H. Lin

**Affiliations:** 1 Department of Radiation Oncology, The University of Texas MD Anderson Cancer Center, Houston, TX, USA; 2 Department of Health Services Research, The University of Texas MD Anderson Cancer Center, Houston, TX, USA

**Keywords:** proton therapy, non–small cell lung cancer, facility type, radiation therapy

## Abstract

**Purpose:**

To examine national care patterns in proton radiation therapy (PBT) use for non–small cell lung cancer (NSCLC) and the effect of facility type on survival.

**Patients and Methods:**

Using the National Cancer Database, we identified 506 patients with a diagnosis of NSCLC from 2004-2014 who underwent PBT. Patients were categorized as having received treatment at an academic/research facility (ARF) or a form of community cancer program (CCP). Descriptive analysis was performed, and overall survival was analyzed by Kaplan-Meier methods and Cox proportional hazard models.

**Results:**

Treatments at ARFs and CCPs were equally distributed with 253 patients at each facility type. A positive trend in PBT use over time was observed with 2.8% of cases being treated in 2008 compared to 21.5% in 2014 (*P* = .001). Definitive doses (≥60 Gy) were more commonly given at ARFs than CCPs (72% versus 45%, respectively; *P* < .001). Five-year overall survival was 31% at ARFs and 18% at CCPs (*P* < .001). On multivariate analysis, outcomes were worse with treatments at CCPs (hazard ratio [HR] 1.61; 95% Confidence Interval, 1.14-2.27; *P* = .007). On subanalysis of nonsurgical patients treated with ≥60 Gy, facility type became insignificant and dose escalation was associated with improved outcomes (≥70 Gy HR 0.45; 95% CI, 0.25-0.81; *P* = .008).

**Conclusion:**

Use of PBT for management of NSCLC is on the rise. Community cancer programs were associated with higher rates of nondefinitive PBT doses and correspondingly worse outcomes. Differences in survival by facility became insignificant when definitive doses were used, warranting further investigation of practice patterns in CCPs at a national level.

## Introduction

Lung cancer remains an aggressive oncologic disease affecting over 220,000 people annually and causing over 25% of all cancer-related deaths in 2017 [1]. Surgical management or definitive radiation therapy (RT) is considered standard of care for early-stage non–small cell lung cancer (NSCLC), while multimodality therapy is preferred for locally advanced disease [2, 3]. In the realm of RT, investigations on the effects of various RT modalities on thoracic malignancies have already led to changes in clinical practice patterns. For example, the utilization of ablative radiation doses through stereotactic body radiation therapy (SBRT) is now considered an acceptable treatment strategy for medically inoperable, stage I NSCLC after randomized clinical trials and retrospective studies demonstrated that SBRT yielded high local control rates and comparable survival outcomes relative to surgery [4, 5]. Photon-based intensity-modulated radiation therapy (IMRT) and volumetric modulated arc therapy have also been shown to provide improved dose sparing of critical structures when compared to conventional 3-dimensional conformal RT.

The application of proton beam therapy (PBT) for treatment of cancer, a concept originally described by physicist Robert Wilson in 1946, has gained traction given the unique physical properties of protons. Compared to photons, which continually deposit dose throughout tissue and exhibit an exit dose, the dose distribution of protons forms a Bragg peak, denoting maximal dose deposition at a finite tissue depth followed by a sharp dose falloff with virtually no exit dose. This dosimetric difference, in turn, can theoretically translate into a reduction of treatment-related morbidity and a beneficial impact on overall survival (OS).

Currently, limited data exist on long-term outcomes of PBT for cancer care. While we await the results of Radiation Therapy Oncology Group (RTOG) 1308, a phase III randomized trial comparing the effects of PBT and photon-based chemoradiotherapy on OS of patients with inoperable stage II-IIIB NSCLC, a recent publication using the National Cancer Database (NCDB) suggests a statistically significantly higher 5-year OS of 22% versus 16% with PBT versus non-PBT, respectively, for patients with stage I-IV NSCLC [6]. The latter retrospective study provided a logistic regression model to determine predictors for receipt of PBT; however, a detailed analysis of the PBT cohort was not provided. Therefore, the objective of our study was to examine the practice patterns of PBT utilization in the United States for the management of stage I-IV NSCLC, with a particular focus on type of treating facility, as this feature has been shown to affect selected management and survival of patients with various cancer types [7–9].

## Materials and Methods

Established in 1989 by the American Cancer Society and the Commission on Cancer (CoC) of the American College of Surgeons, the NCDB is a comprehensive, nationwide clinical surveillance registry with de-identified oncologic data acquired annually from over 1500 CoC-approved centers. Available data include patient demographics, socioeconomic status, tumor characteristics, initial course of therapy, and OS in addition to RT specifics, thereby making the NCDB a valuable resource for patterns of care analyses [10].

**Figure 1.**
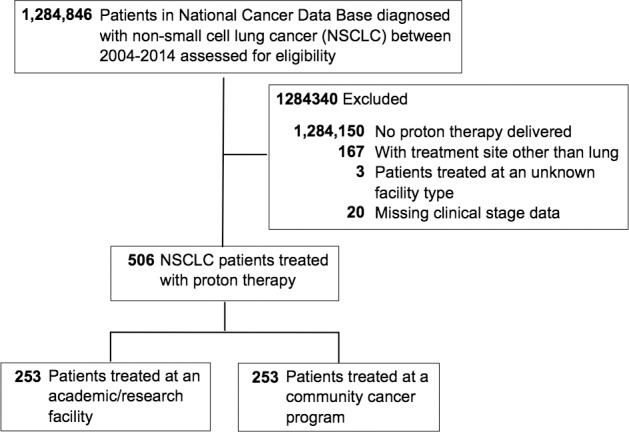
Flow diagram of the study population.

Patients with a diagnosis of clinical stages I-IV NSCLC from 2004 to 2014 were identified. We restricted our study to patients documented as having received PBT to the lungs and/or chest, and stratified our cohort by type of treating facility (Figure 1). Academic/research facilities (ARFs), by definition, are comprehensive cancer centers that treat 500 or more cancer cases annually and participate in postgraduate medical education in at least 4 programs including internal medicine and general surgery. In contrast, community cancer programs (CCPs) must manage 100 to 499 cancer cases annually and are not required to have training programs. Comprehensive CCPs have a similar range of treatment volume and services as ARFs; however, like CCPs, training resident physicians is optional. For the purposes of this study, CCPs (N = 26), comprehensive CCPs (N = 200), and integrated network cancer programs (which require no minimum caseload or resident training, N = 27) were included in the generic “CCP” grouping stratification. Additional reasons for exclusion included unknown clinical stage or facility type, and PBT directed to other sites than the lung and/or chest wall. The timing of RT and surgery was examined and categorized as “no surgery” for presumed definitive RT intent, “preoperative RT,” and “postoperative RT.” Regional doses were then compared among the various RT-surgery sequences and treating facilities.

Baseline patient sociodemographic, clinical, and facility characteristics were compared between ARFs and CCPs by using Pearson X^2^ tests. The primary outcome of interest was OS, defined as the time from diagnosis to last contact or death. Overall survival time for surviving patients was censored at the time of last contact, and the 2-year and 5-year OS by clinical stage and facility type were estimated by using the Kaplan-Meier method. A Cox proportional hazards model was used to study the effects of several factors including facility type on survival, expressed as hazard ratios (HRs). Age, chemotherapy use, and all variables significant on univariable analysis were included in the multivariable analysis (**Supplemental Table 1**). All statistical analyses were performed by using SAS v9.4 software (Cary, North Carolina) and a 2-sided *P* value <.05 was considered statistically significant.

## Results

**Figure 2.**
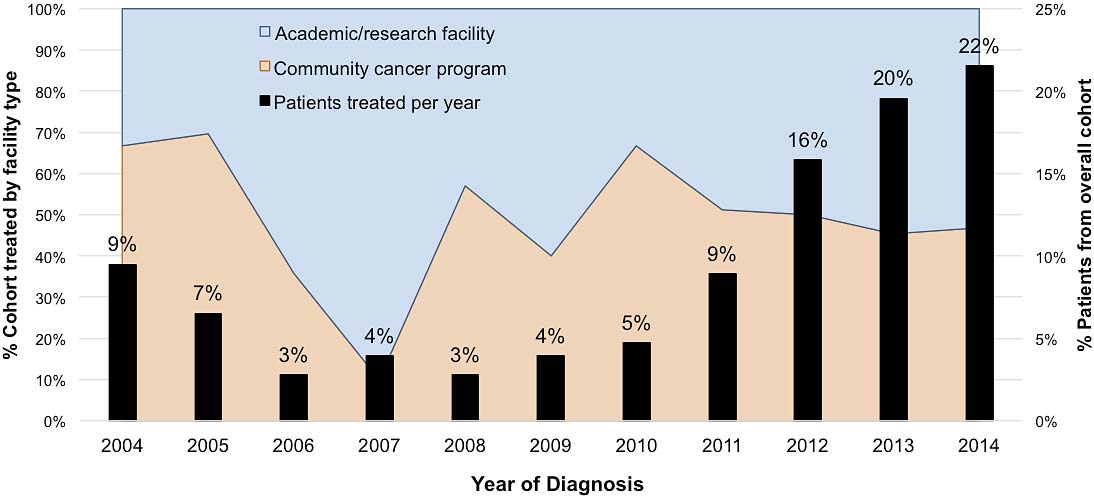
Trends in proton therapy use over time, stratified by facility type. The percentage of patients treated per year is shown as black bars, while the cumulative proportion of patients treated by facility type is reflected in blue and orange.

From 2004 to 2014, a total of 506 patients were given a diagnosis of NSCLC and received PBT. The absolute number of patients treated with PBT fluctuated from 2004-2007; thereafter, a significant positive trend was observed over time with 2.8% of all PBT cases having occurred in 2008 compared to 21.5% in 2014 (N = 14 in 2008 versus N = 109 in 2014; *P* = .011). For the overall cohort, caseload by type of treating facility was evenly distributed with 253 patients treated at an ARF and 253 patients at a CCP. However, this distribution was not consistent on an annual basis until the last 4 years of the study period (**Figure 2**).

### Patient and Clinical Characteristics

The median age was 70 years (range, 42-89 years) and 53% of patients were male. Nearly half of patients were diagnosed with stage III disease (47%), while 25% had stage I, 13% stage II, and 16% stage IV disease. Patients were generally healthy without comorbidities (66%), lived in a zip code with the highest median income quartile ≥$46,000 (47%), and had Medicare (69%). Interestingly, several sociodemographic and clinical features such as gender, median household income, type of insurance, clinical stage, and tumor location were comparable among ARFs and CCPs. However, significant discrepancies existed by facility type with regard to race and comorbidity scores, both of which can be associated with outcomes [11]. Approximately 74% of all black individuals were treated at ARFs, totaling 9% of the ARF population in contrast to 3% of the CCP population (*P* = .004). The ratio of patients with a comorbidity score of 1 and/or ≥2 was disproportionately higher at ARFs (24% and 16%) compared to CCPs (19% and 8%; *P* = .004). On average, patients treated at CCPs lived closer to treating institutions, with a mean and median distance to facility of 29.9 miles and 9.1 miles (range, 0.4-1504 miles) compared to 88 miles and 18.6 miles (range, 0.2-2241.5 miles) for patients treated at ARFs (*P* < .001). Patient and clinical characteristics are shown in **Table 1**.

**Table 1:** General sociodemographic, clinical, and facility characteristics.

**Characteristic**	**Overall, N = 506 (%)**	**ARF, N = 253 (50%)**	**CCP, N = 253 (50%)**	***P*** **value**
Age at diagnosis, y				
Median	70	69	72	.163
Range	42-89	42-89	42-89	
Gender				
Male	270 (53)	131 (52)	139 (55)	.476
Female	236 (47)	122 (48)	114 (45)	
Race				
White	444 (88)	210 (83)	234 (92)	.004
Black	31 (6)	23 (9)	8 (3)	
Other	31 (6)	20 (8)	11 (4)	
Comorbidity score				
0	335 (66)	151 (60)	184 (73)	.004
1	109 (22)	61 (24)	48 (19)	
≥2	62 (12)	41 (16)	21 (8)	
Median household income				
<$30,000 (bottom quartile)	57 (11)	25 (10)	32 (13)	.145
$30K-$34,999	63 (12)	28 (11)	35 (14)	
$35K-$45,000	123 (24)	72 (28)	51 (20)	
≥$46,000 (top quartile)	238 (47)	113 (45)	125 (49)	
Unknown	25 (5)	15 (6)	10 (4)	
Medical insurance				
None/self-pay	3 (1)	2 (1)	1 (0)	.063
Private	116 (23)	51 (20)	65 (26)	
Medicaid	19 (4)	15 (6)	4 (2)	
Medicare	351 (69)	175 (69)	176 (70)	
Other/unknown	17 (3)	10 (4)	7 (3)	
Community type				
Metro	446 (88)	235 (93)	211 (83)	<.001
Urban	32 (6)	3 (1)	29 (11)	
Rural	10 (2)	3 (1)	7 (3)	
Unknown	18 (4)	12 (5)	6 (2)	
Clinical stage				
I	124 (25)	69 (27)	55 (22)	.399
II	65 (13)	29 (11)	36 (14)	
III	238 (47)	119 (47)	119 (47)	
IV	79 (16)	36 (14)	43 (17)	
Primary tumor location				
LUL	139 (27)	79 (31)	60 (24)	.08
LLL	61 (12)	30 (12)	31 (12)	
RUL	149 (29)	74 (29)	75 (30)	
RML	16 (3)	10 (4)	6 (2)	
RLL	70 (14)	35 (14)	35 (14)	
Other/unknown	71 (14)	25 (10)	46 (18)	
Radiation dose range				
<60 Gy	154 (30)	58 (23)	96 (38)	<.001
60-64 Gy	79 (16)	38 (15)	41 (16)	
65-69 Gy	91 (18)	71 (28)	20 (8)	
≥70 Gy	126 (25)	73 (29)	53 (21)	
Unknown	56 (11)	13 (5)	43 (17)	
Chemotherapy				
No chemotherapy	159 (31)	76 (30)	83 (33)	.738
Chemotherapy given	338 (67)	173 (68)	165 (65)	
Unknown	9 (2)	4 (2)	5 (2)	
Surgery				
None	438 (87)	210 (83)	228 (90)	.065
Lobectomy	38 (8)	26 (10)	12 (5)	
Pneumonectomy	7 (1)	5 (2)	2 (1)	
Other/unknown	23 (5)	12 (5)	11 (4)	
Facility location				
Atlantic/New England	177 (35)	126 (50)	51 (20)	<.001
Central	163 (32)	24 (9)	139 (55)	
Pacific/Mountain	166 (33)	103 (41)	63 (25)	
Distance from facility				
≤13 miles	243 (48)	89 (35)	154 (61)	<.001
14-100 miles	201 (40)	121 (48)	80 (32)	
>100 miles	62 (12)	43 (17)	19 (7)	

**Abbreviations**: ARF, academic/research facility; CCP, cancer community program; LUL, left upper lobe; LLL, left lower lobe; RUL, right upper lobe; RML, right middle lobe; RLL, right lower lobe.

### Therapy Specifications Including RT Dosing

With regard to multidisciplinary treatment, 438 patients (87%) did not undergo a surgical intervention. For those that did, lobectomies were more commonly performed (n = 38) than pneumonectomies (n = 7), with 68% (n = 26) of lobectomies performed at ARFs (*P* *=* .065). Approximately two thirds of all patients (n = 338, 67%) received chemotherapy, a proportion that remained roughly equivalent when stratified by facility type (*P* *=* .738). Since total radiation dose delivered can affect survival, we sought to investigate whether differences existed in radiation dosing records between ARFs and CCPs. A higher median dose of 66.6 Gy was delivered at ARFs than the 60-Gy dose at CCPs. According to the National Comprehensive Cancer Network (NCCN), a minimum of 45 Gy in the preoperative setting, 60 Gy with a preferred range of 60 to 70 Gy in the definitive setting (no surgery), and 50 Gy in the postoperative setting are considered standard recommended doses [12]. Compared to national standards, 17% (n = 43) of patients treated at ARFs and 9% (n = 24) of patients treated at CCPs underwent preoperative RT or postoperative RT, most of whom received appropriate radiation doses. However, in the definitive setting, only 43% (n = 108) of patients at CCPs received appropriate doses compared to 66% (n = 168, *P* < .001) at ARFs (**Supplemental Table 2**).

### Survival Analysis

**Figure 3.**
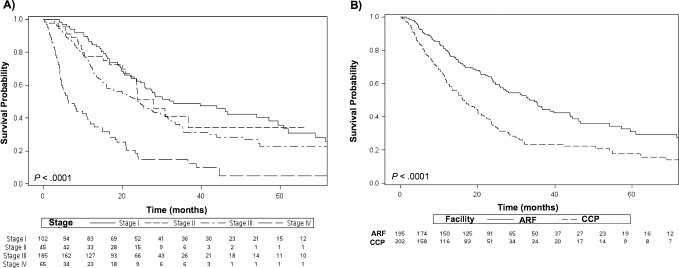
Kaplan-Meier survival analysis by (A) clinical stage and (B) facility type. Abbreviations: ARF, academic/research facility; CCP, cancer community program.

Median follow-up was 15.2 months for the ARF cohort and 23.5 months for the CCP cohort. When stratifying by clinical stage, the 5-year OS was 36% for patients with stage I disease, 34% for stage II, 23% for stage III, and 5% for stage IV (*P* < .001) (**Figure 3A**). The 2-year and 5-year OS estimates for patients treated at ARFs was nearly double that of the CCP population at 61% and 31% compared to 35% and 18%, respectively (*P* < .001) (**Figure 3B**). On multivariate analysis of the overall cohort, receipt of PBT at a CCP was associated with worse outcomes than at ARFs (hazard ratio [HR] 1.61; 95% CI, 1.14-2.27; *P* = .007). Other factors that negatively impacted survival included advanced clinical stage III (HR 2.05; 95% CI, 1.29-3.27; *P* < .003) and stage IV (HR 4.31; 95% CI, 2.62-7.11; *P* < .001), and a lower versus upper lobe tumor location (HR 1.55; 95% CI, 1.11-2.15; *P* = .009). Treatment with chemotherapy (HR 0.5; 95% CI, 0.34-0.74; *P* = .001) or treatments at a facility located in the Atlantic/New England region were associated with improved survival (**Table 2**).

**Table 2:** Multivariate results for the overall cohort and nonsurgical cohort that received definitive RT doses (≥60 Gy).

**Overall cohort**				**Definite RT cohort, nonsurgical**		
**Variable**	**HR (95% CI)**		***P*** **value**	**HR (95% CI)**		***P*** **value**
Facility type						
ARF	1			1		
CCP	1.61	(1.14-2.27)	0.007	1.7	(0.97-2.99)	.066
Age at diagnosis, y						
≤50	1			1		
51-60	0.91	(0.39-2.15)	0.837	0.82	(0.21-3.24)	.781
61-70	0.94	(0.43-2.06)	0.872	1.24	(0.35-4.38)	.735
71-80	1.27	(0.57-2.8)	0.561	1.4	(0.39-5.03)	.603
≥81	1	(0.42-2.36)	0.999	1.52	(0.4-5.78)	.536
Radiation dose range^a^						
<60 Gy	1			-	-	-
60-64 Gy	1.18	(0.77-1.8)	0.459	1		
65-69 Gy	1.45	(0.87-2.42)	0.159	1.47	(0.7-3.1)	.306
≥70 Gy	0.63	(0.41-0.95)	0.027	0.45	(0.25-0.81)	.008
Surgery						
No surgery	1			-	-	-
Lobectomy	0.46	(0.21-1)	0.051	-	-	-
Pneumonectomy	1.64	(0.47-5.68)	0.439	-	-	-
Other/unknown	1.48	(0.79-2.74)	0.218	-	-	-
Chemotherapy						
No chemotherapy	1			1		
Chemotherapy given	0.5	(0.34-0.74)	0.001	0.75	(0.37-1.53)	.431
Unknown	0.68	(0.22-2.11)	0.503	3	(0.33-26.94)	.327
Clinical stage						
I	1			1		
II	1.25	(0.69-2.28)	0.463	1.42	(0.59-3.4)	.432
III	2.05	(1.29-3.27)	0.003	1.92	(0.88-4.21)	.103
IV	4.31	(2.62-7.11)	<0.001	4.97	(1.82-13.53)	.002
Primary tumor location						
Upper lobe	1			1		
Middle lobe	1.03	(0.45-2.34)	0.95	1.29	(0.34-4.88)	.711
Lower lobe	1.55	(1.11-2.15)	0.009	1.28	(0.78-2.08)	.329
Other	1.01	(0.67-1.53)	0.954	1.08	(0.56-2.07)	.818
Facility location						
Atlantic/New England	1			1		
Central	1.98	(1.29-3.04)	0.002	2.85	(1.34-6.04)	.006
Pacific/Mountain	1.67	(1.04-2.66)	0.033	2.36	(1.07-5.19)	.033
Year of diagnosis						
2014	1			1		
2013	0.67	(0.43-1.04)	0.073	0.68	(0.36-1.28)	.23
2012	1.5	(0.96-2.33)	0.073	1.48	(0.78-2.79)	.23
2011	1.18	(0.67-2.08)	0.572	1.91	(0.74-4.91)	.181
2010	1.03	(0.51-2.06)	0.941	1.26	(0.43-3.68)	.67
2009	2.17	(1.05-4.5)	0.037	1.7	(0.57-5.1)	.342
2008	0.86	(0.36-2.02)	0.723	1.06	(0.34-3.31)	.919
2007	1.96	(1.01-3.77)	0.045	2.56	(1.03-6.36)	.043
2006	1.19	(0.55-2.58)	0.666	1.08	(0.29-4.06)	.910
2005	1.4	(0.79-2.48)	0.251	0.99	(0.36-2.69)	.976
2004	1.44	(0.81-2.57)	0.216	1.72	(0.66-4.45)	.267

a For the Radiation dose range variable, patients treated with “< 60 Gy” were excluded from the multivariate analysis of the “definitive RT cohort, nonsurgical”.

**Abbreviations**: RT, radiation therapy; HR, hazard ratio; CI, confidence intervals; ARF, academic/research facility; CCP, cancer community program.

Given that most PBT patients did not undergo surgery, we performed a subset analysis including only nonsurgical patients who received ≥60 Gy. When definitive RT doses were used, the type of treating facility became insignificant (CCP HR 1.7; 95% CI, 0.97-2.99; *P* = .066). Moreover, dose escalation ≥70 Gy was significantly associated with improved outcomes over 60 to 64 Gy (HR 0.45; 95% CI, 0.23-0.81; *P* = .008) (**Table 2**).

## Discussion

Although RT has been an integral part of the paradigm of cancer treatment for many decades, the application of PBT in the field has been a rather recent development. Loma Linda University Medical Center (Loma Linda, California) opened the first hospital-based proton center in 1990 and to date, there are 25 operating centers nationwide with an estimated 11 centers under construction [13, 14]. The dose-sparing attributes of PBT relative to other RT modalities make it an ideal treatment modality for various diagnoses including pediatric cancers and cancers of the prostate, head and neck, or lung. Indeed our study, which demonstrates an absolute annual increase in the number of patients with NSCLC being treated with PBT, correlates with the positive global trend [15, 16]. Apart from physical access, type of insurance and finances can often affect the access patients have to proton therapy. In our study, 69% of patients had Medicare and 23% were privately insured. However, those underrepresented were patients who were uninsured/self-pay (1%) or on Medicaid (4%). In a similar manner, only 23% (n = 120) of patients pertained to the lowest 2 median household income quartiles, compared to 71% (n = 361) of patients making $35,000 or more per year. Therefore, efforts should be directed towards addressing these socioeconomic disparities, particularly if future NSCLC clinical trials demonstrate a survival benefit with PBT over current photon-based treatments [17].

Our study provides survival outcomes by hospital type, which has been recommended as a useful quality metric of cancer care [18]. Type of treating facility has been shown to affect long-term outcomes of patients with various cancer types. For example, for patients with oral cavity cancer, Rubin et al [9] found that being treated at an ARF was associated with a higher likelihood of undergoing surgical intervention and improved outcomes than treatment at a CCP (HR 0.95; 95% CI, 0.91-0.98; *P* < .01). Furthermore, Dholaria et al [19] noted that treatment at ARFs was associated with higher 4-year OS than at CCPs (29% versus 22%; *P* < .001) for all patients with NSCLC. In the present study, we found that in a generally well-balanced cohort, with the exception of comorbidity score and ethnicity, which favored the CCP population, 5-year OS was significantly higher at ARFs than CCPs (31% versus 18%; *P* < .001). On multivariate analysis, treatment at a CCP remained significantly associated with worse outcomes (HR 1.61; 95% CI, 1.14-2.27; *P* = .007). Another potential difference in patient care by facility type that may influence long-term outcomes is the available salvage therapies offered after recurrences are detected. We were unable to investigate this in our current study; therefore, additional studies are required to assess modern practice patterns in the salvage setting and how they vary in the community compared to academic institutions.

Hospital volumes-outcome relationships have been well documented for surgical interventions [20–22]; however, we chose to analyze the cohort by facility type instead as a surrogate for multiple known and unknown factors that can influence treatment selection. Both ARFs and CCPs must report a caseload of 500 or more cancer cases annually; however, only ARFs are required to participate in postgraduate medical education programs and are likely to also have additional resources. Using the Surveillance, Epidemiology and End Results (SEER)–Medicare database, Charlton et al [7] discovered that institutions with National Cancer Institute (NCI) designation or affiliation with residency programs and/or major medical schools were more likely to provide guideline-concordant care to patients with stage II/III rectal cancer than institutions lacking these qualifications. Similarly, the use of recommended neoadjuvant chemotherapy for select patients with breast cancer has been shown to occur less frequently at CCPs than ARFs [23], raising awareness of notable practice pattern variations.

One pertinent distinction in treatments between facilities was that CCPs more frequently delivered lower total radiation doses than ARFs. Not only was the median dose lower at 60 Gy compared to 66.6 Gy, but the ratio of patients receiving an underdose at <60 Gy in the definitive RT setting was 2.5 times greater at CCPs (32% versus 13%). Rationalization for this observation can be multifactorial, including early termination of treatment due to treatment-related toxicities, patient noncompliance, lack of resources for management of severe toxicities, or a general practice trend towards offering preoperative doses with the expectation of surgical intervention thereafter. Errors in reported radiation doses are also plausible; however, this is less likely as the NCDB has strict registry documentation procedures similar to the SEER database to audit and correct data if needed, and it is unlikely that CCPs across the nation are consistently having similar documentation errors relative to ARFs.

Radiation dose is known to affect local regional control and survival. When taking definitive dosing into account in our subanalysis, the discrepancy in outcomes between ARFs and CCPs became insignificant, indicating that RT dose remains a predictor of survival and that dose delivery variations among facility types warrant further investigation at an institutional and national level. Interestingly as with other studies [24–26], we observed a survival benefit with doses ≥70 Gy (HR 0.45; 95% CI, 0.25-0.81; *P* = .001). NCCN guidelines currently recommend definitive RT doses between 60 to 70 Gy with caution of doses up to 74 Gy given the findings of RTOG 0617, which found no difference in survival between 60 Gy and 74 Gy for patients with inoperable stage III NSCLC [3, 27]. However, this trial allowed for only 3-dimensional conformal RT or IMRT, both of which are photon-based modalities and may lead to higher doses to thoracic structures such as the heart, esophagus, and spinal cord in comparison to PBT. Therefore, additional studies are needed to determine whether higher radiation doses delivered through PBT can yield improved survival without an increase in the treatment toxicity profile.

While facility type became insignificant in our multivariate subanalysis, the location of the treating facility remained a significant predictor of survival, with patients treated in the Atlantic/New England region having improved outcomes as compared to patients who received their care in Central and Pacific/Mountain locations. Part of the discrepancy can be associated with a higher proportion of ARFs to CCPs located in the Atlantic/New England region (71% versus 29%) compared to the Central (15% versus 85%) and Pacific/Mountain (62% versus 38%) locations. However, even after accounting for type of treating facility, these findings with respect to facility location suggest that regional practice patterns may have a larger impact on survival than facility type. Differences in primary management of patients with NSCLC by geographic location should therefore be further investigated to determine potential socioeconomic, demographic, and clinicopathologic factors associated with the observed differences in treatments and outcomes.

The question of whether PBT offers a survival benefit over photon-based NSCLC treatments is currently being investigated by the phase III randomized trial RTOG 1308. In the interim, a recent publication using the NCDB compared outcomes of 243,474 patients with NSCLC of all clinical stages to those of 348 patients who received photon-based RT (non-PBT) or PBT, respectively [6]. In their analysis, the authors reported a significant 5-year OS difference of 22% with PBT compared to 16% with non-PBT. However, when they stratified photon-based treatments by modality, there was no survival improvement with PBT relative to IMRT (HR 1.05; 95% CI, 0.91-1.20; *P* = .524). Radiation dose ranges were also captured in their study, but the authors did not further elucidate on reasons why the dose range was broad (0.01-1395.6 Gy) and whether accounting for definitive doses ≥60 Gy would affect the OS benefit seen with PBT over general photon-based RT. The present study, which analyzes a similar PBT cohort from 2004-2014, differs from the previous study as it reflects a more thorough analysis on PBT and demonstrates the impact of facility type on outcomes for patients with NSCLC. Moreover after noticing a survival difference between ARFs and CCPs, we were able to identify RT dose variations by facility type as a probable cause.

Several limitations exist in the current study, given its retrospective nature. We are unable to account for the extent of primary disease that was targeted with PBT or what quality assurance measures were in place. A detailed analysis regarding dose variations (such as hypofractionated or stereotactic body RT regimens) by facility type and clinical stage was attempted but not reported given significant variations in available fractionation data. Therefore, total delivered RT dose with a 60-Gy cutoff for definitive dosing was used, since most patients had stage II or III disease (60%) and would likely require conventional dosing over more aggressive dose per fractionation schemes owing to larger treatment field sizes. Further specifications regarding PBT, such as passive versus active scanning delivery methods, are unavailable in the NCDB and therefore could not be analyzed. Information regarding the type(s) of chemotherapy used or the number of cycles given is also unavailable in the NCDB. Moreover, compared to other NCDB studies analyzing outcomes using photon-based treatments for NSCLC, the size of the overall PBT cohort is relatively small at 506 patients. However, this is the largest study to date and having pooled from over 70% of the cancer population in the United States, it provides relevant findings on PBT practice patterns and associated outcomes as compared to what could be achieved at a single or multi-institutional level.

## Conclusion

In summary, the application of PBT for management of NSCLC is becoming more common over time. For unknown reasons, patients receiving treatment at ARFs were more likely to receive guideline-concordant care with respect to appropriate radiation doses and had better OS than patients treated at CCPs. When examining nonsurgical patients treated with recommended RT doses of ≥60 Gy, the effect of facility type on survival became insignificant, warranting an evaluation of practice patterns and outcomes at an institutional level, particularly in community center programs. Lastly, given the dose-sparing advantages of protons over photons, clinical trials should examine the role of dose escalation using PBT for NSCLC to determine if a dose-outcomes relationship with limited toxicity exists.

## References

[1] Siegel RL, Miller KD, Jemal A (2017). Cancer statistics, 2017. *CA Cancer J Clin*.

[2] Rigas JR, Kelly K (2007). Current treatment paradigms for locally advanced non-small cell lung cancer. *J Thorac Oncol*.

[3] Ettinger DS, Wood DE, Chair Fred V, Aisner DL, Bauman J, Ross Camidge D, Chirieac LR, D TA, DeCamp MM, Dilling TJ, Dobelbower M, Govindan R, Gubens MA, Hennon M, Komaki R, Lacker RP, Michael Lanuti P, Leisch LJ, Lin J, Loo BW, Martins R, Otterson GA, Reckamp K, Riely GJ, Schild SE, Shapiro TA, Stevenson J, Swanson SJ, Tauer K, Jude S, Yang SC, Kristina Gregory N, Miranda Hughes O, Buffet Cancer Center (2018). NCCN Guidelines Version 2.2018 Panel Members Non-Small Cell Lung Cancer-Farber/Brigham and Women's Cancer Center.

[4] Timmerman R, Paulus R, Galvin J, Michalski J, Straube W, Bradley J, Fakiris A, Bezjak A, Videtic G, Johnstone D, Fowler J, Gore E, Choy H (2010). Stereotactic body radiation therapy for inoperable early stage lung cancer. *JAMA*.

[5] Chang JY, Senan S, Paul MA, Mehran RJ, Louie AV, Balter P, Groen HJM, McRae SE, Widder J, Feng L, van den Borne BEEM, Munsell MF, Hurkmans C, Berry DA, van Werkhoven E, Kresl JJ, Dingemans A-M, Dawood O, Haasbeek CJA, Carpenter LS, De Jaeger K, Komaki R, Slotman BJ, Smit EF, Roth JA (2015). Stereotactic ablative radiotherapy versus lobectomy for operable stage I non-small-cell lung cancer: a pooled analysis of two randomised trials. *Lancet Oncol*.

[6] Higgins KA, O’Connell K, Liu Y, Gillespie TW, McDonald MW, Pillai RN, Patel KR, Patel PR, Robinson CG, Simone CB, Owonikoko TK, Belani CP, Khuri FR, Curran WJ, Ramalingam SS, Behera M (2017). National Cancer Database analysis of proton versus photon radiation therapy in non-small cell lung cancer. *Int J Radiat Oncol*.

[7] Charlton ME, Hrabe JE, Wright KB, Schlichting JA, McDowell BD, Halfdanarson TR, Lin C, Stitzenberg KB, Cromwell JW (2016). Hospital characteristics associated with stage II/III rectal cancer guideline concordant care: analysis of Surveillance, Epidemiology and End Results-Medicare data. *J Gastrointest Surg*.

[8] Chu QD, Zhou M, Peddi P, Medeiros KL, Zibari GB, Shokouh-Amiri H, Wu X-C (2017). Influence of facility type on survival outcomes after pancreatectomy for pancreatic adenocarcinoma. *HPB (Oxford)*.

[9] Rubin SJ, Cohen MB, Kirke DN, Qureshi MM, Truong MT, Jalisi S (2017). Comparison of facility type outcomes for oral cavity cancer: analysis of the national cancer database. *Laryngoscope*.

[10] Bilimoria KY, Stewart AK, Winchester DP, Ko CY (2008). The National Cancer Data Base: a powerful initiative to improve cancer care in the United States. *Ann Surg Oncol*.

[11] Shavers VL (2002). Racial and ethnic disparities in the receipt of cancer treatment. *Cancer Spectrum Knowl Environ*.

[12] Ettinger DS, Wood DE, Chair Fred V, Aisner DL, Bauman J, Ross Camidge D, Chirieac LR, D TA, DeCamp MM, Dilling TJ, Dobelbower M, Govindan R, Gubens MA, Hennon M, Komaki R, Lackner RP, Michael Lanuti P, Leisch LJ, Lin J, Loo BW, Martins R, Otterson GA, Reckamp K, Riely GJ, Schild SE, Shapiro TA, Stevenson J, Swanson SJ, Tauer K, Jude S, Yang SC, Kristina Gregory N, Miranda Hughes O, Buffett Cancer Center NCCN Guidelines Version 6.2018.

[13] Proton therapy - The National Association for Proton Therapy (NAPT) proton beam therapy, protons and prostate cancer (2018). http://www.proton-therapy.org/.

[14] (2018). Proton radiation therapy treatment history. https://protons.com/proton-advantage/history-proton-radiation-therapy.

[15] Jermann M (2015). Particle therapy statistics in 2014. *Int J Part Ther*.

[16] Sisterson J (2005). Ion beam therapy in 2004. https://ac.els-cdn.com/S0168583X0501308X/1-s2.0-S0168583X0501308X-main.pdf?_tid=c31d54f4-050f-11e8-9b7a-00000aab0f27&acdnat=1517242769_9a07f5563bfccec95aaf6a3bb6dc2050.

[17] RTOG (2018). Clinical Trials | Study Number 1308.

[18] Shulman LN, Palis BE, McCabe R, Mallin K, Loomis A, Winchester D, McKellar D (2018). Survival as a quality metric of cancer care: use of the National Cancer Data Base to assess hospital performance. *J Oncol Pract*.

[19] Lou Y, Dholaria B, Soyano A, Hodge D, Cochuyt J, Manochakian R, Ko SJ, Thomas M, Johnson MM, Patel NM, Miller RC, Adjei AA, Ailawadhi S (2018). Survival trends among non-small-cell lung cancer patients over a decade: impact of initial therapy at academic centers. *Cancer Med*.

[20] Birkmeyer JD, Siewers AE, Finlayson EV, Stukel TA, Lucas FL, Batista I, Welch HG, Wennberg DE (2002). Hospital volume and surgical mortality in the United States. *N Engl J Med*.

[21] Rogers SO, Ayanian JZ, Ko CY, Kahn KL, Zaslavsky AM, Sandler RS, Keating NL (2009). Surgeons' volume of colorectal cancer procedures and collaborative decision-making about adjuvant therapies. *Ann Surg*.

[22] Schmidt CM, Turrini O, Parikh P, House MG, Zyromski NJ, Nakeeb A, Howard TJ, Pitt HA, Lillemoe KD (2010). Effect of hospital volume, surgeon experience, and surgeon volume on patient outcomes after pancreaticoduodenectomy. *Arch Surg*.

[23] Mohiuddin JJ, Deal AM, Carey LA, Lund JL, Baker BR, Zagar TM, Jones EL, Marks LB, Chen RC (2016). Neoadjuvant systemic therapy use for younger patients with breast cancer treated in different types of cancer centers across the United States. *J Am Coll Surg*.

[24] Kong F-M, Ten Haken RK, Schipper MJ, Sullivan MA, Chen M, Lopez C, Kalemkerian GP, Hayman JA (2005). High-dose radiation improved local tumor control and overall survival in patients with inoperable/unresectable non-small-cell lung cancer: long-term results of a radiation dose escalation study. *Int J Radiat Oncol Biol Phys*.

[25] Rengan R, Rosenzweig KE, Venkatraman E, Koutcher LA, Fox JL, Nayak R, Amols H, Yorke E, Jackson A, Ling CC, Leibel SA (2004). Improved local control with higher doses of radiation in large-volume stage III non-small-cell lung cancer. *Int J Radiat Oncol Biol Phys*.

[26] Machtay M, Bae K, Movsas B, Paulus R, Gore EM, Komaki R, Albain K, Sause WT, Curran WJ (2012). Higher biologically effective dose of radiotherapy is associated with improved outcomes for locally advanced non-small cell lung carcinoma treated with chemoradiation: an analysis of the Radiation Therapy Oncology Group. *Int J Radiat Oncol Biol Phys*.

[27] Bradley JD, Paulus R, Komaki R, Masters G, Blumenschein G, Schild S, Bogart J, Hu C, Forster K, Magliocco A, Kavadi V, Garces YI, Narayan S, Iyengar P, Robinson C, Wynn RB, Koprowski C, Meng J, Beitler J, Gaur R, Curran W, Choy H (2015). Standard-dose versus high-dose conformal radiotherapy with concurrent and consolidation carboplatin plus paclitaxel with or without cetuximab for patients with stage IIIA or IIIB non-small-cell lung cancer (RTOG 0617): a randomised, two-by-two factorial phase 3 study. *Lancet Oncol*.

