# Supplementary material for: Trends and Outcomes of Proton Radiation Therapy Use for Non–Small Cell Lung Cancer
Source: Int J Part Ther. 2018 Nov 30;5(2):18–27. doi: 10.14338/IJPT/18-00029.1 (PMC6874194; doi:10.14338/IJPT/18-00029.1)
Supplement: Supplementary file 1 [file ijpt-05-02-06_s01.docx]

**Supplemental Table 1.** Univariate analysis for the overall cohort

| **Variable** | **HR (95% CI)** | | **P value** |
| --- | --- | --- | --- |
|  |  |  |  |
| **Facility type** |  |  |  |
| ARF | 1 |  |  |
| CCP | 1.88 | (1.45-2.43) | <0.001 |
| **Age at diagnosis, years** |  |  |  |
| ≤ 50 | 1 |  |  |
| 51-60 | 1.33 | (0.63-2.83) | 0.456 |
| 61-70 | 1.128 | (0.57-2.25 | 0.732 |
| 71-80 | 1.51 | (0.76-3.01) | 0.238 |
| ≥ 81 | 1.448 | (0.69-3.04) | 0.327 |
| **Gender** |  |  |  |
| Male | 1 |  |  |
| Female | 0.91 | (0.70-1.17) | 0.456 |
| **Race** |  |  |  |
| White | 1 |  |  |
| Black | 0.87 | (0.52-1.45) | 0.602 |
| Other | 0.72 | (0.42-1.21) | 0.212 |
| **Comorbidity** **score** |  |  |  |
| 0 | 1 |  |  |
| 1 | 1.06 | (0.78-1.45) | 0.718 |
| ≥ 2 | 1.04 | (0.69-1.57) | 0.860 |
| **Radiation dose range** |  |  |  |
| ≥50 Gy | 1 |  |  |
| <50 Gy | 2.23 | (1.64-3.04) | <0.001 |
| **Surgery** |  |  |  |
| No surgery | 1 |  |  |
| Lobectomy | 0.29 | (0.14-0.58) | <0.001 |
| Pneumonectomy | 0.99 | (0.32-3.09) | <0.001 |
| Other/Unknown | 1.14 | (0.66-1.96) | 0.221 |
| **Chemotherapy** |  |  |  |
| No chemotherapy | 1 |  |  |
| Chemotherapy given | 0.79 | (0.61-1.03) | 0.084 |
| Unknown | 1.15 | (0.42-3.12) | 0.788 |
| **Clinical stage** |  |  |  |
| I | 1 |  |  |
| II | 1.01 | (0.60-1.71) | 0.960 |
| III | 1.24 | (0.90-1.70) | 0.193 |
| IV | 2.98 | (2.04-4.34) | <0.001 |
| **Primary tumor location** |  |  |  |
| Upper lobe | 1 |  |  |
| Middle lobe | 1.04 | (0.48-2.22) | 0.925 |
| Lower lobe | 1.45 | (1.07-1.96) | 0.016 |
| Other | 1.49 | (1.05-2.10) | 0.025 |
| **Facility location** |  |  |  |
| Atlantic/New England | 1 |  |  |
| Central | 2.18 | (1.54-3.10) | <0.001 |
| Pacific/Mountain | 1.88 | (1.35-2.63) | <0.001 |
| **Year of diagnosis** |  |  |  |
| 2014 | 1 |  |  |
| 2013 | 0.71 | 0.46-1.10) | 0.126 |
| 2012 | 1.4 | (0.91-2.17) | 0.126 |
| 2011 | 1.57 | (0.95-2.58) | 0.076 |
| 2010 | 1.1 | (0.57-2.12) | 0.771 |
| 2009 | 1.42 | (0.74-2.73) | 0.296 |
| 2008 | 0.98 | (0.47-2.05) | 0.961 |
| 2007 | 1.94 | (1.09-3.44) | 0.024 |
| 2006 | 1.53 | (0.77-3.05) | 0.223 |
| 2005 | 1.81 | (1.09-3.01) | 0.022 |
| 2004 | 1.92 | (1.22-3.02) | 0.005 |
| **Medical insurance** |  |  |  |
| Private | 1 |  |  |
| None/Self-Pay | 0.97 | (0.30-3.12) | 0.952 |
| Medicaid | 1.25 | (0.64-2.45) | 0.514 |
| Medicare | 1.1 | (0.81-1.49) | 0.532 |
| Other/Unknown | 0.98 | (0.45-2.16) | 0.968 |
| **Community type** |  |  |  |
| Metro | 1 |  |  |
| Other | 1.25 | (0.84-1.84) | 0.27 |
| **Median household income** |  |  |  |
| < $30,000 (bottom quartile) | 1 |  |  |
| $30K - $34,999 | 0.74 | (0.45-1.22) | 0.234 |
| $35K - $45,000 | 0.72 | (0.46-1.11) | 0.132 |
| ≥ $46,000 (top quartile) | 0.77 | (0.52-1.14) | 0.188 |
| **Distance from facility** |  |  |  |
| ≤13 miles | 1 |  |  |
| 14-100 miles | 0.78 | (0.59-1.02) | 0.07 |
| >100 miles | 0.7 | (0.46-1.06) | 0.93 |

Abbreviations: ARF, academic/research facility; CCP, cancer community program; RT, radiation therapy.

**Supplemental Table 2.** Radiation therapy dosing by treatment sequence and facility type

| **Patients treated at ARFs** | | | | | |
| --- | --- | --- | --- | --- | --- |
| RT-Surgery sequence | < 45 Gy | 45-59 Gy | ≥ 60 Gy | NOS | *Total (col %)* |
|  |  |  |  |  |  |
| No surgery | 12 (4.7) | 21 (8.3) | 168 (66.4) | 9 (3.6) | **210 (83)** |
| Preoperative RT | 0 (0) | 10 (4) | 0 (0) | 0 (0) | **10 (4)** |
| PORT | 1 (0.4) | 13 (5.1) | 14 (5.5) | 5 (2) | **33 (13)** |
| *Total (row %)* | **13 (5)** | **44 (17)** | **182 (72)** | **14 (6)** | **253 (100)** |
| **Patients treated at CCPs** | | | | | |
| RT-Surgery sequence | < 45 Gy | 45-59 Gy | ≥ 60 Gy | NOS | *Total (col %)* |
|  |  |  |  |  |  |
| No surgery | 44 (17.4) | 37 (14.6) | 108 (42.7) | 40 (15.8) | **228 (91)** |
| Preoperative RT | 1 (0.4) | 2 (0.8) | 2 (0.8) | 1 (0.4) | **6 (2)** |
| PORT | 4 (1.6) | 8 (3.2) | 4 (1.6) | 2 (0.8) | **18 (7)** |
| *Total (row %)* | **49 (19)** | **47 (19)** | **114 (45)** | **43 (17)** | **253 (100)** |

Abbreviations: ARF, academic/research facility; CCP, cancer community program; PORT, postoperative radiotherapy; RT, radiation therapy.
